# The effect of specialized Hongquan martial arts training on the development of visual-motor integration in children

**DOI:** 10.3389/fpsyg.2025.1598526

**Published:** 2025-10-09

**Authors:** Juan-Juan Zhang, Jia Lu, Chen-Tao Liu

**Affiliations:** ^1^Department of Physical Education, Xi’an International Studies University, Xi’an, Shaanxi, China; ^2^Department of Physical Education, Northwest University, Xi’an, Shaanxi, China

**Keywords:** VMI, spatial relationships, postural balance, inhibitory control, long-term exercise, traditional martial arts

## Abstract

**Objective:**

This study examines the effects of different durations of Hongquan martial arts training on the development characteristics of visual-motor integration (VMI) abilities in children and the relevant influencing factors.

**Methods:**

A total of 59 children, aged 9–11 years, who were active practitioners of Hongquan martial arts were recruited from the Lu Jia Hongquan Cultural Heritage Base and the Xiaolong Martial Arts School in Xi’an. Additionally, 21 children of the same age were recruited from a Primary School in Hi-Tech District, Xi’an, as the normal control group. The subjects were divided into four groups based on their years of training: the no-training group, 1-year training group, 3 years training group, and 5+ years training group. The visual-motor integration ability, visual perception ability, action coordination ability, attention, and executive function of the subjects were tested using the Beery-Buktenica developmental test of visual-motor integration (VMI-6), Test of Visual Perception Skills 4th edition (TVPS-4), Motor coordination evaluation index system, Children Attention Test, Flanker task, more-odd shifting task, and 1-back task, respectively.

**Results:**

Compared to the no-training group, the one-year Hongquan martial arts practitioners showed significant improvement in working memory updating function (*p < 0.05*), and the three-year Hongquan martial arts practitioners showed significant improvement in balance ability (*p < 0.01*); those with five or more years of Hongquan martial arts training showed significant improvements in their VMI abilities (*p < 0.01*), particularly in visual perception (*p < 0.05*), spatial relationships (*p < 0.01*), overall coordination (*p < 0.05*), balance ability (*p < 0.01*), attention breadth (*p < 0.01*), and inhibitory control (*p < 0.05*). In addition, the 5 + years of training group showed significantly higher levels of balance ability and overall coordination ability than the group with 1 year of Hongquan martial arts training (*p < 0.05*), and better overall coordination ability and attention allocation than the group with 3 years of Hongquan martial arts training (*p < 0.05*). However, the working memory updating function of the 3-years and 5+ years groups was significantly lower compared to the 1-year group (*p < 0.05*).

**Conclusion:**

Long-term Hongquan martial arts training for more than 5 years during the sensitive period for the development of VMI abilities can effectively enhance the visual-motor integration abilities of children.

## 1 Introduction

Visual-motor integration (VMI) is the ability of an individual to integrate and coordinate visual perceptions with fine motor skills during purposeful activities. It is an important factor influencing the quality of an individual’s activity processes and level of motor skills, and is strongly related to the development of children’s skills in areas such as mathematics (Flores et al., 2024), drawing, and handwriting (Cheong et al., 2024). In addition, poor visual-motor integration ability is a common performance characteristic of children with developmental coordination disorder, attention deficit hyperactivity disorder (ADHD), and autism spectrum disorder (ASD). It is clear that there is an urgent need to study effective intervention methods to improve visual-motor integration ability in children.

Multiple factors influence VMI ability, including visual perception ability, motor coordination ability, executive function, attention, etc. (Fang et al., 2017). Visual perception is one of the two main components of VMI ability, encompassing visual recognition and discrimination, visual memory, visuospatial orientation and processing, graphical perception, etc. Sequential memory is known to be a mediator of the relationship between visual-motor integration and self-care performance (Lin et al., 2022). Research by Pierre Vereecken has found that spatial perception in early childhood is an important part of visual perception. As another important component of VMI skills, motor coordination is related to the reconstruction of mental representations during the visual-motor integration process (Beery, 2010). Fine motor coordination is thought to have a reciprocal relationship with the development of VMI, and the development of VMI proceeds in step with the development of fine motor skills in children. The performance of VMI tasks not only involves coordination of motor control with visual perception, but also complex cognitive processes such as planning, task flexibility, goal orientation, response inhibition, and maintaining attention throughout the task (Kobayashi-Cuya et al., 2018; Takarae et al., 2007). Studies have suggested that executive function may be closely related to VMI (Cameron et al., 2015). Research by Becker et al. (2014) has shown that inhibitory control and working memory are significantly correlated with visual-motor skills in preschool and kindergarten children, but studies to date on the correlation between cognitive flexibility and VMI have been limited. Furthermore, the brain regions involved in the transmission of visual information in the dorsal pathway have been found to overlap with those involved in attention control (Atkinson, 2017). This suggests that attention control plays a crucial role in the VMI process (Wang et al., 2024). Currently, many studies have focused on different factors related to VMI skills, but visual perception ability, motor coordination, executive function, and attention control have only been studied in isolation.

All the processes that can directly affect VMI, such as visual perception (i.e., spatial and attentional skills), fine motor coordination, and gross motor skills, are known to be plastic and trainable (Diamond and Lee, 2011; Uttal et al., 2013). For example, there is evidence that visual training programs can significantly improve visual perception abilities and VMI skills in students with visual impairments (Abduljaber et al., 2022). Krüger et al. (2009) found that provincial-level cricket players under the age of 19 years showed improvements in VMI after participating in an eight-week visual skills training program. The Rhythmic Motor and Behavioral Intervention program produced medium-to-large improvements in gross motor skills, including stationary and locomotor abilities, visual-motor integration, and balance (Cheong et al., 2024). The development of VMI skills is highly sensitive and dynamic, with rapid growth occurring between the ages of 4–7 years (Decker et al., 2011), peaking between 4 and 5 years old (Fang et al., 2017), but continuing to develop at least until the age of 12 years. Most studies have shown that the effectiveness of long-term interventions of at least 7 months for improving VMI skills (Kadar et al., 2020), but 12 sessions of treatment within 3 months are also important for improving VMI. Therefore, it is crucial to exercise and develop this skill during the critical age of rapid development of VMI.

Hongquan is a traditional Chinese martial art with a long history dating back to the Zhou and Qin dynasties, developing into a relatively complete set of routines during the Tang and Song dynasties. Hongquan martial arts are renowned for their rich content and comprehensive techniques, covering many aspects such as disc (basic skills), method (techniques and methods), potential (postures and forms), and principle (martial principles). It has also evolved into the “Seven Systems of One Fist” (i.e., Hongquan, Paoquan, Huaquan, Jiuquan, Zuiquan, Tongbei, and Ziquan), which are deeply loved by a wide audience. The techniques of Hongquan martial arts can be summarized as “Sustaining and Chopping as the Foundation, Hooking and Hanging as the Ability, Gripping and Striking as the Method, and Transforming the Body as the Marvel.” “Sustaining and Chopping as the Foundation” means that blocking and striking are the most fundamental techniques, encompassing the eight core methods of fighting. “Hooking and Hanging as the Ability” refers to the use of movements like Cloud Hands (alternating circular movements with both hands) and Sweeping Hands (lightly wiping or blocking with the hands) to neutralize and redirect the opponent’s blows. Hooking involves changing from one technique to another, while hanging involves deflecting the force of the opponent’s attack, constantly seeking opportunities amidst the flux of combat. “Gripping and Striking as the Method” involves observing the opponent’s movements and striking suddenly with gripping techniques to neutralize their force, then swiftly retracting after the strike. “Transforming the Body as the Marvel” emphasizes bodily agility, requiring practitioners to master shoulder extension, arm rotation, waist twisting, and hip pivoting. These movements ensure fluidity in the movements of the fists and feet, using evasion and displacement to weaken the opponent’s force before seizing opportunities for counterattack. These techniques are characterized by swift eyes and agile feet, with coordinated movements that fully demonstrate the successful integration and coordination of visual perception and motor coordination.

Exercise, as a special form of stimulation, can positively impact children’s cognitive control (Chaddock-Heyman et al., 2013; Chang et al., 2016; Lu et al., 2018), motor coordination (Irie et al., 2021), visual skills (Krzepota et al., 2015), and motor performance. As a form of exercise intervention, Hongquan martial arts are distinct from other sports due to their notable characteristics, including “deceitful striking and clever hitting, flexible body movements, fierce leg techniques, and comprehensive grappling skills.” Practicing Hongquan martial arts requires fluid movements and wholehearted devotion to the art. In practical combat, the use of eight different fighting techniques involves constant changes in hand movements, emphasizing the principle of “where the hands go, the eyes follow” by which the line of sight should continuously follow the movement of the hand, keeping the brain engaged to maintain clear perception of the opponent’s movements. This hand-eye coordination ability is a critical component of martial arts training, which has different effects on individuals with different exercise years. Given Hongquan martial arts involve unique movement patterns and have unique technical characteristics, but there has been a lack of research on how such training could improve VMI ability and cognitive performance during adolescent growth and development. Thus, exploring the use of Hongquan martial arts training interventions to maintain and enhance human brain structure and function presents an exciting avenue for research. Thus, this study investigates the development characteristics of visual-motor integration abilities in children with varying years of Hongquan martial arts training, with the goal of providing important evidence for effective interventions targeting VMI improvement.

## 2 Materials and methods

### 2.1 Participant characteristics

In this study, a total of 80 children aged 9–11 years with varying durations of Hongquan martial arts training [no-training group: 21 participants (16 male, 10.12 ± 1.34 years), 1-year training group: 20 participants (17 male, 9.68 ± 0.54 years), 3-years training group: 20 participants (16 male, 10.17 ± 0.78 years), and 5 + years training group: 19 participants (15 male, 10.69 ± 1.21 years)] were recruited from a primary school in Gaoxin District, the Lu Jia Chinese Hongquan Cultural Inheritance Base, and the Xiaolong Martial Arts School in Xi’an to participate in the experiment. The inclusion criteria were as follows: all participants were right-handed, had no cardiovascular or cerebrovascular diseases, no physical disabilities, normal or corrected-to-normal visual acuity, no color blindness or other color weakness, normal intelligence, and were familiar with basic computer operations. In addition, they had not previously participated in any similar studies. Before the start of the study, the parents gave informed consent for their children’s participation. This study was approved by the Ethics Committee of Xi’an International Studies University.

### 2.2 Exercise program

Each training session was based on physical fitness activities for children, commencing with 5 min of neural activation exercises followed by a 10 min static and dynamic stretching warm-up. Next, fundamental Hongquan martial arts training in was conducted, encompassing leg techniques and basic footwork combinations. Building upon this foundation, students systematically learned basic Hongquan martial arts routines, such as the Hongquan Thirteen Forms and the Ten Major Stances. The final stage involved Hongquan martial arts combat practice, in which participants engaged in live sparring using unarmed techniques alongside weaponry such as knives and swords, all conducted with professional protective gear.

Instructors dynamically adjusted the training intensity and exercise volume based on the participants’ actual learning progress and comprehension of martial arts movements, ensuring that the training process was scientific, safe, and effective. The exercise program was scheduled for two sessions per week, each lasting 120 min. It comprehensively employed circuit training, repetition training, interval training, and the principle of progressive overload to enhance participants’ physical conditioning and technical proficiency. Class sizes ranged from 15 to 20 participants. Both instructors hold certifications in children’s physical training and physical fitness instruction, as well as fifth-degree black belts or higher in Chinese martial arts. They each possess over a decade of extensive experience in Hongquan martial arts instruction and training. In addition, both instructors had undertaken two or three specialized training courses annually focused on teaching Hongquan martial arts to young people, thereby continually enhancing their teaching capabilities.

### 2.3 Procedure

#### 2.3.1 Visual-motor integration ability test

The Beery-Buktenica Developmental Test of Visual-Motor Integration, Sixth Edition was used to assess the visual-motor integration ability of the participants. The VMI test consists of 30 geometric figures, ranked from easy to difficult. The participants are required to copy each image into the space below the figure as accurately as possible in terms of shape and size. Each figure is copied once, and no erasing or altering is allowed. During the test, the participant is required maintain proper posture, and the test booklet should be placed parallel on the desktop without being moved randomly. There is no time limit for performing the test. The raw score is calculated by counting the number of correctly reproduced figures, with each figure worth one point. The raw scores are then converted to standard scores according to the participant’s age, and statistical analysis is performed.

#### 2.3.2 Visual perception ability test

The Test of Visual Perception Skills Fourth Edition (TVPS-4) was used to measure seven dimensions of visual perception: Visual Discrimination (DIS), Visual Memory (MEM), Spatial Relationships (SPA), Form Constancy (CON), Sequential Memory (SEQ), Visual Figure-Ground (FGR), and Visual Closure (CLO). Each sub-test comprises 18 items, with each item counting for one point. The final score for each sub-test is converted to a standard score based on the participant’s age, and the sub-scores and total scores are then analyzed.

#### 2.3.3 Motor coordination ability test

Motor coordination ability was assessed using the evaluation criteria developed by Zhang (2021), based on seven indicators: reaction ability, spatial orientation ability, proprioception ability, rhythm ability, balance ability, cognitive ability related to motor actions, and overall coordination ability. Specifically, reaction ability was evaluated by using the hexagonal rebound test, with each participant tested three times and the highest score taken as the final result. Spatial orientation ability was evaluated using the cross-quadrant turn-and-jump test, in which participants jump continuously for 30 s and the correct number of jumps is recorded as the score, with each participant tested twice and the best result taken as the final score. Proprioceptive ability was evaluated using the one-minute rope skipping test, with the number of skips in 1 min recorded as the score. Rhythm ability was evaluated using the bar return test, with the time required to complete the task recorded as the score, with each participant tested twice and the best result taken as the final score. Balance ability was evaluated using the eight-point star-shaped offset balance test, in which dynamic balance ability is assessed in three directions: anterior medial, medial, and posterior medial. Each participant was tested twice in each direction, with the highest of the two scores taken as the final score for that direction, and the final overall score calculated as the average of the three directions. Cognitive ability related to motor actions was evaluated using the rope and ladder recognition test, with the time taken for accurate completion taken as the score. For the shuttle jump exercise, each error was calculated as a 0.5 s penalty to their score, with each participant tested twice and the best result taken as the final score. Overall coordination ability was evaluated using the back and forth traverse test, with the number of crosses within 20 s recorded as the score, and with each participant tested twice and the best result taken as the final score.

#### 2.3.4 Executive function test

Executive function was assessed using three sub-tasks on a laptop computer (14-inch screen, resolution 1,366 × 768), and all programs were written using E-prime 2.0 software. Before each task, the operator first guided the participant through a practice session. Formal testing began only when the practice accuracy rate reached ≥ 80%.

The specific tasks for each of the three subfunctions are as follows:

The flanker task was used to evaluate inhibitory control (Hillman et al., 2006). In the experimental task, the flanker paradigm was divided into consistent conditions (“FFFFF” “LLLLL”) and inconsistent conditions (“LLFLL” “FFLFF”). The task required participants to respond to the middle letter as quickly as possible while ensuring accuracy, pressing the “F” key if it was “F,” and pressing the “L” key if it was “L.” The formal test consisted of two blocks, with each block comprising 48 trials. Prior to the formal test, participants completed 12 practice trials. The subject’s test results were calculated as:


$$\begin{array}{l} \text{Inhibitory reaction time}=\\ (\text{inconsistent reaction time}-\text{consistent reaction time}). \end{array}$$

The more-odd shifting task was used to evaluate switching function (Kiesel et al., 2010). The experimental materials were composed of “the digits 1–9, excluding 5,” and there were three judgment requirements in the test:

1. When black numbers were presented, participants were required to judge the relationship between the presented numbers and 5, pressing the “F” key if the number was less than 5, and pressing the “J” key if the number was greater than 5.
2. When green numbers were presented, participants were required to judge the odd-even nature of the presented numbers, pressing the “F” key if the number was odd, and pressing the “J” key if it was even.
3. When alternating between black and green numbers were presented, the task shifted between judgement of size (black numbers) and of odd-even (green numbers), requiring participants to make corresponding color response keys. The formal test consisted of six blocks presented in an ABCCBA sequence, in which blocks A and B required no task-switching required and consisted of 16 trials each, and block C was the task-switching condition, consisting of 32 trials (including 16 switch trials). Prior to the first A/B block, participants completed eight practice trials; before block C, they completed 16 practice trials. The subject’s test results were calculated as:

$$\text{Switching reaction time}=[\begin{array}{l} \text{shifting reaction time}-\\ (\begin{array}{l} \text{size reaction time}+\mathrm{odd}-\\ \text{even reaction time} \end{array})/2 \end{array}].$$

A 1-back task was used to evaluate working memory updating function (Jaeggi et al., 2016). In the experimental task, the experimental stimuli were 20 English letters. Each letter was presented alone in the center of the computer screen, and participants were required from the second letter to judge whether the current letter was the same as the previous one, pressing the “F” key if they were the same and the “J” key if they were different. The formal test consisted of two blocks, with each block comprising 20 trials. Prior to the formal test, the participants completed 12 practice trials. The average reaction time was taken as the final test result; a shorter time is taken to indicate better refreshing ability.

#### 2.3.5 Attention test

The Adolescents Attention Test developed by Heng-Chan Yin was used to measure the children’s abilities for attention shifting, attention stability, attention breadth, and attention allocation. Specifically, the figure discrimination test was used to evaluate attention allocation, participants were required to identify a target figure from a set of 300 circular ring figures arranged in 20 rows and 15 columns within a specified time of 3 min, with the number of correct answers recorded as the score. The four-circle test was used to evaluate attention breadth: participants were required to identify the block containing four circles out of 650 blocks arranged in 25 rows and 26 columns with different numbers of circles within a specified time of 3 min, with the number of correct responses recorded as the score. The visual tracking test was used to evaluate attention stability: participants were required to use their eyes to track 35 curves with the starting point on the left and the ending point on the right within a specified time of 2 min, and to write the sequence number at the beginning of the row into the square at the end of the curve on the right side. The number of correct responses was recorded as the score. The addition-subtraction test was used to evaluate attention shifting: participants were required to perform alternating addition and subtraction operations on adjacent numbers within a specified time and write the operation results in the middle position between two adjacent numbers. Each group of numbers consisted of 22 numbers from 1 to 9, there was a total of 12 groups, and the allotted test time was 3 min. The number of correct responses was recorded as the score.

### 2.4 Statistical analysis

The data results are presented here as mean ± standard deviation (M ± SD). The statistical analysis software SPSS 22.0 was used to analyze the experimental data. One-way ANOVA was used to compare and analyze the different factors affecting visual-motor integration ability in children with different years of Hongquan martial arts training. Post-hoc pairwise comparisons were performed using Tukey’s test. The significance level of statistical analysis was set at *p < 0.05*, and the level of extreme significance was *p < 0.01*.

## 3 Results

### 3.1 Comparison and analysis of the characteristics of the development of the visual-motor integration ability of children with different durations of Hongquan martial arts training

The results of one-way ANOVA show that the children who had trained in Hongquan martial arts for five or more years had significantly higher visual-motor integration ability than those with no training (*F = 4.252*, *p < 0.01*). There were no significant differences in visual-motor integration abilities between no training group and either the 1-year or 3-years groups, suggesting that the development of visual-motor integration ability through Hongquan martial arts training in children requires long-term practice to for significant improvement to occur (see Table 1).

**Table 1:** Development characteristics of visual-motor integration ability in children with different durations of Hongquan martial arts training.

Test indicators	No Hongquan training group	1-Year Hongquan training group	3-Years Hongquan training group	5 + Years Hongquan training group
VMI	93.94 ± 3.17	98.25 ± 9.08	95.62 ± 8.54	102.54 ± 6.89**

VMI: Visual-Motor Integration; **p < 0.01, compared to the no Hongquan training group.

### 3.2 Analysis of results for the impact of different durations of Hongquan martial arts training on visual perception ability

To assess changes in the influencing factors related to the visual-motor integration ability of children who had trained in Hongquan martial arts for different lengths of time, we further conducted a statistical analysis of the characteristics of the changes in their visual perception ability and found that the standard scores for the visual perception ability of the 5 + years training group were significantly higher than those of the no training group (*F = 3.632*, *p < 0.05*). Of these, the spatial relationship ability in particular was significantly increased (*F = 4.622*, *p < 0.05*), whereas increases in visual discrimination, visual memory, form constancy, sequential memory, visual figure-ground, and visual closure were not statistically significant (*F* = 2.271, 1.184, 0.739, 0.662, 1.337, and 0.460, respectively) (see Table 2).

**Table 2:** Comparison of visual perception ability and sub-ability scores in children with different durations of Hongquan martial arts training.

Test indicators	No Hongquan training group	1-Year Hongquan training group	3-Years Hongquan training group	5+ Years Hongquan training group
Visual perception ability	72.76 ± 8.27	72.75 ± 8.33	75.39 ± 6.13	81.54 ± 8.26*
DIS	10.65 ± 2.18	10.38 ± 2.83	10.23 ± 1.79	12.15 ± 1.68
MEM	10.47 ± 1.81	10.63 ± 1.19	10.85 ± 1.63	11.38 ± 1.33
SPA	9.12 ± 2.18	9.75 ± 1.28	10.23 ± 1.54	11.46 ± 1.61**
CON	10.76 ± 2.41	10.63 ± 2.33	10.77 ± 1.69	11.85 ± 2.12
SEQ	11.18 ± 2.43	11.13 ± 1.55	12.31 ± 1.65	12.00 ± 2.20
FGR	9.94 ± 1.82	9.88 ± 2.42	10.31 ± 1.65	11.54 ± 2.07
CLO	10.65 ± 2.81	10.25 ± 1.58	10.69 ± 1.55	11.23 ± 1.74

*p < 0.05, compared to the no Hongquan training group.

### 3.3 Analysis of the results for the impact of different durations of Hongquan martial arts training on motor coordination ability

Comparative analyses of the seven indicators of motor coordination ability revealed that the overall coordination ability and balance ability for the 5 + years training group were significantly higher than those for the no training group (*F = 4.097*, *p < 0.05*; *F = 8.213*, *p < 0.01*) and 1-year training group (*p < 0.05*, *p < 0.05*). In addition, the balance ability of the children in the 3-years training group was significantly higher than that of those in the no training group (*p < 0.01*). The overall coordination ability of children in the 5 + years group was also significantly higher than that of those in the 3-years training group (*p < 0.05*), but there were no significant differences in the other indicators of motor coordination ability (see Table 3).

**Table 3:** Comparison of motor coordination ability and sub-ability scores in children with different durations of Hongquan martial arts training.

Test indicators	No Hongquan training group	1-Year Hongquan training group	3-Years Hongquan training group	5+ Years Hongquan training group
Overall coordination ability (times)	17.41 ± 3.12	16.63 ± 4.98	17.46 ± 3.73	22.18 ± 5.23*&#
Reaction ability (points)	2.76 ± 0.75	1.63 ± 1.30	2.15 ± 1.21	2.27 ± 1.27
Proprioception (times)	105.06 ± 21.70	105.38 ± 27.19	107.69 ± 28.79	124.18 ± 32.77
Rhythm ability (s)	7.04 ± 1.01	7.21 ± 1.06	7.46 ± 2.69	6.97 ± 1.10
Spatial orientation ability (times)	23.47 ± 6.26	27.13 ± 4.85	22.00 ± 8.46	26.55 ± 10.38
Cognitive ability related to motor actions (s)	26.18 ± 7.05	25.89 ± 14.41	20.98 ± 12.57	19.15 ± 7.74
Balance ability (cm)	76.75 ± 5.91	78.99 ± 6.41	87.82 ± 7.52**	90.73 ± 12.93**&

*p < 0.05, **p < 0.01, compared to the no Hongquan training group; &*p* < 0.05, compared to the 1-year Hongquan training group, #*p* < 0.05, compared to the 3-years Hongquan training group.

### 3.4 Analysis of results for the impact of different durations of Hongquan martial arts training on attention

As can be seen in Table 4, the attention test data indicate that the attention allocation score of children in the 5 + years group was significantly higher than that of those in the 3-years training group (*F = 2.527*, *p < 0.05*), and the attention breadth score of children in the 5 + years group was significantly higher than that of those in the no training group (*F = 6.165*, *p < 0.01*). There were no statistical differences in attention stability or attention shifting among the four groups.

**Table 4:** Comparison of attention levels in children with different durations of Hongquan martial arts training.

Test indicators	No Hongquan training group	1-Year Hongquan training group	3-Years Hongquan training group	5+ Years Hongquan training group
Attention allocation (figure discrimination)	12.59 ± 8.02	10.63 ± 11.01	8.46 ± 5.59	17.91 ± 10.39#
Attention breadth (find four circles)	61.12 ± 9.21	53.88 ± 13.04	67.00 ± 10.37	76.27 ± 16.49**
Attention stability (visual tracking)	10.94 ± 3.21	10.13 ± 4.73	11.31 ± 2.63	13.36 ± 3.36
Attention shifting (addition-subtraction)	41.76 ± 22.99	45.63 ± 19.23	52.54 ± 29.25	66.91 ± 26.91

**p < 0.01, compared to the no Hongquan training group; #*p* < 0.05, compared to the 3-years Hongquan training group.

### 3.5 Analysis of results for the impact of different durations of Hongquan martial arts training on executive function

In terms of changes in executive functions, the inhibitory control of the children in the 5 + years group (*F = 2.410*, *p < 0.05*) and the working memory updating function of those in the 1-year training group (*F = 3.892*, *p < 0.05*) were significantly better than those for the no training group. However, a significant prolongation of the effect of refreshing reaction time was observed in children in the 3-years and 5 + years groups compared to those in the 1-year training group (*F = 3.892*, *p < 0.05*). There were no statistically significant differences in the change of switching function among any of the groups (see Table 5).

**Table 5:** One-way ANOVA results of executive function development characteristics in children with different durations of Hongquan martial arts training.

Sub-function	No Hongquan training group	1-Year Hongquan training group	3-Years Hongquan training group	5+ Years Hongquan training group
Inhibitory control reaction time difference (ms)	158.69 ± 180.29	113.62 ± 63.81	106.15 ± 82.97	33.06 ± 51.61*
Switching function reaction time difference (ms)	396.37 ± 261.96	142.22 ± 412.08	275.74 ± 400.97	362.18 ± 205.06
Working memory updating function reaction time (ms)	496.03 ± 121.68	347.79 ± 164.07*	509.51 ± 77.98&	512.93 ± 91.41&

*p < 0.05, compared to the no Hongquan training group; &*p* < 0.05, compared to the 1-year Hongquan training group.

## 4 Discussion

This study compared and analyzed the differences in visual-motor integration ability and related influencing factors in children practicing Hongquan martial arts for different durations. The findings indicate that children with five or more years of Hongquan martial arts training had significantly higher scores in visual-motor integration ability than those in the 1-year and 3-years training groups. This effect was mainly manifested in improvements in sub-abilities such as visual perception ability, spatial relationships, overall coordination ability, balance ability, attention breadth, and inhibitory control. This suggests that the continuous tracking and synchronization of one’s own movements with the rapidly changing techniques during Hongquan martial arts training strengthen the visuomotor neural circuitry, thereby promoting the development of visual-motor integration ability in children through long-term training.

There are known to be significant age differences in children’s visual-motor integration ability. Specifically, VMI ability develops rapidly before children are 5 years old, develops in complex ways under the influence of educational factors when they are 5–7 years old (Cong, 2020), continues to develop steadily when they are 8–10 years old, and remains stable after 11–12 years old (Carsone et al., 2021; Heiz and Barisnikov, 2016). In this study, Hongquan martial arts training for five or more years was found to significantly enhance the children’s visual-motor integration ability. Further analysis of the age distribution of the participants revealed that those in the 5 + years of training group started practicing Hongquan martial arts at the ages of 5–6 years, while the majority of the 3-years training group started at age seven, and the 1-year training group mainly started between ages 8–10. This covariation between starting age and accumulated training suggests that early initiation of training may influence VMI development by extending the duration of effective training. However, the hierarchical regression did not detect any statistically significant independent effects for either starting age or training duration. Future studies should employ longitudinal designs that control for starting age to further clarify the underlying mechanisms involved.

Visual perception ability is the primary factor affecting visual-motor integration ability and thus plays a crucial role in its development. Visual–spatial processing is a prerequisite for successfully maintaining visual-motor integration stability. Research by Abduljaber et al. (2022) has shown that visual training programs can significantly improve the visual perception ability and VMI skills of students with visual impairments. In the current study, children practicing Hongquan martial arts had to flexibly apply eight different techniques (e.g., sustain, chop, hook, hang, bind, block, stick, and carry), constantly changing their hand movements while tracking their hands with their eyes. These activities engaged their brains and thus promoted the development of their visual perception ability, which is consistent with the results of Abduljaber’s research. Similarly, during Hongquan sparring practice, the emphasis on offensive and defensive awareness and adaptability required the children to interact with their peers through complex visual and auditory systems, thus enhancing their understanding and perception of spatial relationships, and thereby improving their visual–spatial abilities. Research by Dou (2007) similarly found that five or more years of systematic martial arts training can significantly improve athletes’ visual–spatial relationship abilities. As a form of martial arts, Hongquan martial arts have significant effects on the exercise and improvement of visual–spatial abilities during the training process. This is reflected not only in routine practice but also in actual combat, further confirming the positive impact of martial arts training on the improvement of visual–spatial ability. It is worth noting that other sub-abilities such as visual discrimination and memory did not show significant improvement; this may be related to the fact that Hongquan martial arts emphasize dynamic spatial processing rather than static visual analysis (e.g., rapidly tracking moving targets in actual combat).

Contemporary children and adolescents (particularly the alpha generation, born between 2011 and 2025, i.e., those under age 14) are digital natives whose behavioral patterns are profoundly shaped by technological trends and online information. This exposes them to multifaceted developmental risks (Ziatdinov and Cilliers, 2022). The proliferation of short videos and instant information has shortened attention spans (Lad, 2024), and inappropriate technology use heightens the risk of physical health problems (Chen and Nakagawa, 2023). In addition, physical fitness deficiencies have become prominent, with widespread declines in motor skills and reduced leisure-time physical activity (Orhan et al., 2024). These factors may have potential implications for VMI development, which is built upon a foundation of complete visual perception, fine motor coordination, motor inhibition, and sustained attention (Ahn, 2021). The core factors affecting the development of visual motor integration ability are different at different ages. For children aged 4–6, motor coordination ability and cognitive flexibility are the primary influencing factors of visual-motor integration ability. Recent systematic reviews have suggested that occupational therapy interventions for children aged 0–5 years can promote their motor development, which is an important component of visual-motor integration ability (Tanner et al., 2020). This study extends Tanner’s research by demonstrating that 3 years of Hongquan martial arts training significantly enhances balance ability in children, and five or more years of training significantly improves both their overall coordination ability and their balance abilities. Zhang Kang et al. found that task-oriented training can significantly improve the balance ability of children with developmental coordination disorder, enhance their hand-eye coordination, and improve their fine motor skills, speed, and agility. Moreover, these the results had strong stability (Zhang et al., 2018). Balance is the ability of the human body to maintain body posture under dynamic or static conditions (Lai et al., 2022). Postural adjustment and body balance require the coordination of the vestibular, proprioceptive, and visual sensory organs, which transmit information about acceleration and spatial displacement to the central nervous system. The CNS analyzes and integrates these pieces of information and then transmits the resulting outputs through the vestibulospinal pathway to effectors to regulate muscle tension, relaxation, and balance control (Taubert et al., 2011). Chinese martial arts have high requirements for posture control. During practice, the external body parts (hands, eyes, trunk, and feet) must move in harmony as a whole, while internal elements (spirit, will, breath, and strength) must be highly unified. “External training” requires perfect coordination of hand, eye, body, and step and other body postures, such as hand-eye coordination and trunk and foot coordination. “Internal training” requires internal unity of mind, spirit, offensive and defensive shifts, and breath, thereby ensuring coordination between breath and strength, and between mind and breath (Wang, 2008). Compared with conventional physical activities, Chinese martial arts emphasize the overall external coordination of the body and a high degree of internal mental concentration, and thus training in Chinese martial arts can effectively promote the development of children’s vestibular and proprioceptive senses and other sensory functions. In addition, martial artists tend to have stronger upper limb motor control and coordination ability, possibly due to the high degree of coordination of their overall movements, which promotes the development of proprioception and reduces the delay time in generating biomechanical force from upper limb muscles (Alesi et al., 2014; Ma et al., 2018). Martial arts training also enhances explosive strength and improves flexibility and reaction speed, and this may be an important factor in the improvement in the balance ability in preschool children achieved through training in Chinese martial arts (Fukuda et al., 2011; Sekulic et al., 2006).

Cognitive flexibility impacts visual-motor integration ability, involving both working memory and inhibitory control components, and is more of a mixed executive function that uses inhibitory control to make it possible to flexibly change the content of working memory. Fang et al. used the Beery VMI to confirm the relationships linking motor coordination, visual perception, executive function, and VMI skills in preschool children aged 4–6 years (Fang et al., 2017). The current study extends those findings, showing that long-term Hongquan martial arts training significantly improves inhibitory control in children. The study by Yan (2016) found that after 14 weeks of martial arts instruction, the experimental group showed significant improvements in accuracy and response time on the Stroop test. In addition, a few researchers have found that working memory significantly impacts visual-motor integration ability, suggesting that executive function and visual-motor integration share overlapping neural networks, such as the cerebellum and basal ganglia. In the current study, the children’s 1-back reaction time was found to be significantly shorter after short-term practice of Hongquan martial arts, but significantly longer after medium- and long-term practice. This phenomenon may reflect the dual effects of training intensity on cognitive function: short-term training reduces working memory load through motor automation (Kahneman, 1973), while long-term high-intensity training may offset this advantage due to increased neural signal noise. According to Kahneman’s attention resource allocation model, this change suggests that as training intensity increases, the cognitive resources originally spared through automation may later be reallocated to motor control, leading to a decline in cognitive task performance.

Visual spatial attention develops from infancy to childhood, and the related inhibitory control ability is essential for the development of children’s attention network. This study found that long-term practice of Hongquan martial arts for more than 5 years promotes the development of children’s attention breadth. Prior studies have shown that scientific practice of martial arts, from each individual movement or the entire routine, can promote the development of children’s brains and improve their attention ability. Notably, the study by Wu (2005) found that physical exercise had a significant impact on the attention breadth of fifth-grade primary school students. The study by Li et al. (2010) similarly found that martial arts routine training had a significant effect of improving children’s attention breadth, and the study by Yan and Yang (2005) found that martial arts could improve children’s attention and reaction ability. The results of this study indicate that, in long-term practice of Hongquan martial arts, the two factors of inhibitory control and attention breadth play an important role in the development process of children’s visuomotor integration ability.

In summary, this study comprehensively explores the impact of different durations of Hongquan martial arts training on visual-motor integration ability and the relevant influencing factors involved. However, as a preliminary exploratory study in this field, it has some limitations worth noting, as they provide possible directions for future research. First, this study is primarily focused on the assessment of behavioral indicators and did not examine the underlying mechanisms by which the training produced its effects. Future studies could use neuroimaging to explore the neural mechanisms and plasticity involved in cognitive training. Second, this study used a cross-sectional design to compare groups with different training durations, but this approach is not as effective as longitudinal studies in establishing causality. Future studies could adopt longitudinal tracking designs to more accurately assess the effects of training and further control for the influence of potential confounding variables such as socioeconomic status, school environment, and extracurricular activities. Third, the potential effect of gender on the development of visual-motor integration ability was not analyzed in the study. There may be differences between males and females in terms of motor ability and cognitive function, and future studies could further explore the moderating role of gender in the effects of Hongquan martial arts training. Finally, future studies could compare the effects of Hongquan martial arts versus other sports (e.g., ball games/gymnastics) on VMI to further validate its unique benefits.

## 5 Conclusion

This study explored the changing characteristics of the effects of different durations of Hongquan martial arts training on visual-motor integration ability and a combination of factors that can directly affect visual-motor integration ability. The following conclusions were drawn: Hongquan martial arts training for five or more years had a more positive impact on children’s visual-motor integration ability compared to 3 years or 1 year of practice. This effect was mainly reflected in improvements in visual perception ability (especially spatial relationships), motor coordination ability (specifically overall coordination ability and balance ability), attention breadth, and inhibitory control in executive function. Hongquan martial arts training for 3 years and five or more years has an adverse effect on the working memory updating function. This may be due to the appearance of a large amount of neural signal noise with the increased exercise intensity, which could have led to a decline in individual cognitive performance. The development of visual-motor integration abilities in children exhibits a sensitive period. As a form of physical intervention, Hongquan martial arts training significantly enhances visual-motor integration ability, and its effects are closely correlated with the age at which practice begins and the duration of practice.

## Data Availability

The raw data supporting the conclusions of this article will be made available by the authors, without undue reservation.
